# Central and nephrogenic diabetes insipidus: updates on diagnosis and management

**DOI:** 10.3389/fendo.2024.1479764

**Published:** 2025-01-08

**Authors:** Kathryn Flynn, Jennifer Hatfield, Kevin Brown, Nicole Vietor, Thanh Hoang

**Affiliations:** ^1^ Department of Internal Medicine, Walter Reed National Military Medical Center, Bethesda, MD, United States; ^2^ Department of Endocrinology, Walter Reed National Military Medical Center, Bethesda, MD, United States

**Keywords:** diabetes insipidus, central diabetes insipidus, nephrogenic diabetes insipidus, polyuria, polydipsia, copeptin, diagnosis, treatment

## Abstract

Diabetes insipidus (DI) is a rare endocrine disease involving antidiuretic hormone (ADH), encompassing both central and nephrogenic causes. Inability to respond to or produce ADH leads to inability of the kidneys to reabsorb water, resulting in hypotonic polyuria and, if lack of hydration, hypernatremia. DI cannot be cured and is an unfamiliar disease process to many clinicians. This diagnosis must be distinguished from primary polydipsia and other causes of hypotonic polyuria. The main branchpoints in pathophysiology depend on the level of ADH pathology: the brain or the kidneys. Prompt diagnosis and treatment are critical as DI can cause substantial morbidity and mortality. The gold standard for diagnosis is a water deprivation test followed by desmopressin administration. There is promising research regarding a new surrogate marker of ADH called copeptin, which may simplify and improve the accuracy in diagnosing DI in the future. Patients with DI require adequate access to water, and there are nuances on treatment approaches depending on whether a patient is diagnosed with central or nephrogenic DI. This article describes a stepwise approach to recognition, diagnosis, and treatment of DI.

## 1 Introduction

Diabetes insipidus (DI) is a rare endocrine disease in which the kidney is unable to concentrate urine, either due to the lack of antidiuretic hormone (ADH) or an inability to respond to ADH. This leads to excess excretion of hypotonic urine. DI affects 1 in 25,000 people, can develop at any age, and is seen equally in males and females (1, 2). Morbidity and mortality are not routinely reported in DI as this disease primarily affects quality of life. One exception is in patients with adipsic DI, which is central DI that in which the patient lacks a thirst sensation. Though exceedingly rare with only a couple hundred cases reported in the medical literature, roughly 40% of cases have occurred following treatment of a ruptured anterior communicating artery aneurysm. When patients are unable to sense their thirst, they are prone to severe dehydration that subsequently increases their mortality. Reports also show risks of hyponatremia, venous thromboembolism, seizures, acute kidney injury, rhabdomyolysis, anterior pituitary hormone deficiencies, and sleep-disordered breathing (3, 4).

**Figure 1:**
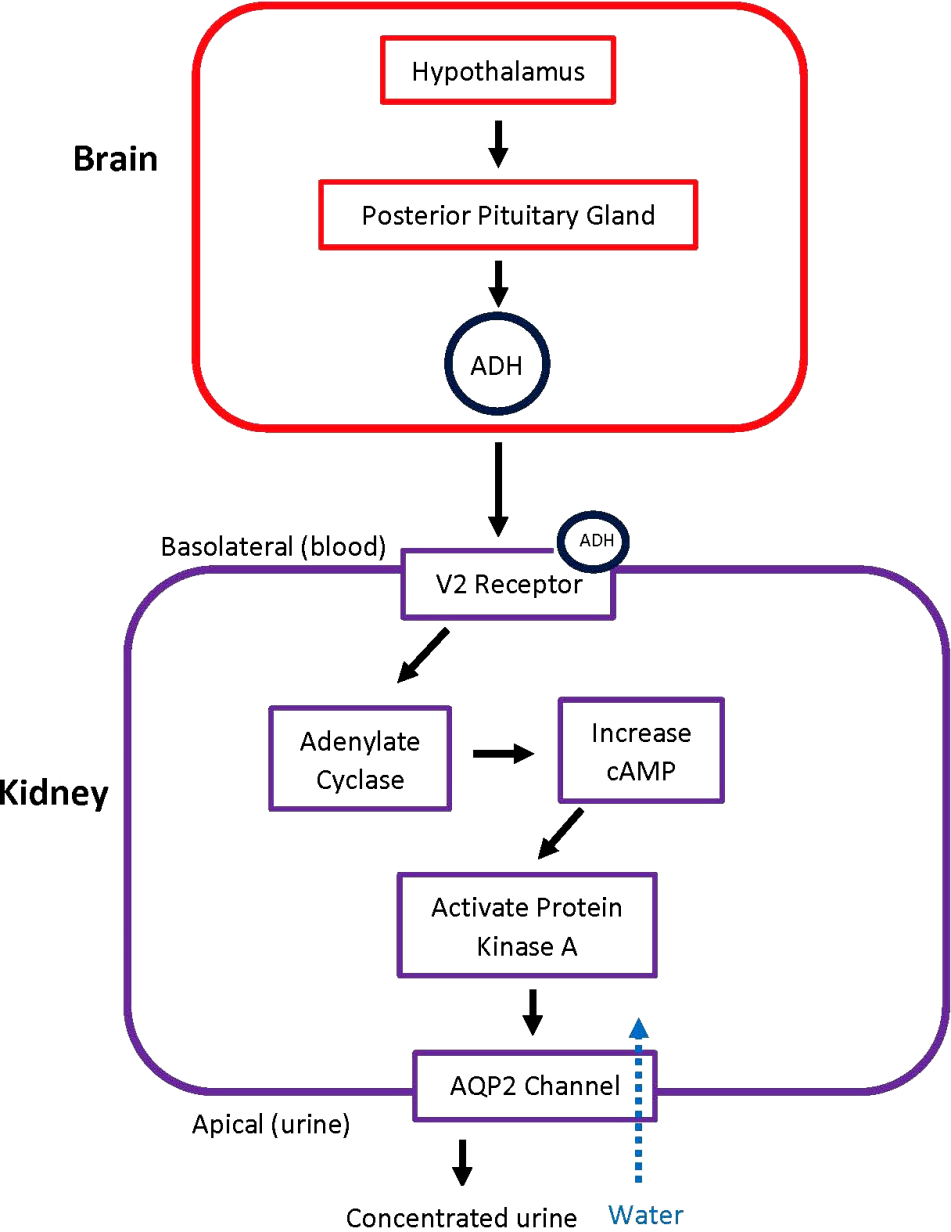
Pathway of ADH Release and Action. When there is a trigger, the hypothalamus signals the posterior pituitary gland to release ADH. ADH then acts on V2 receptors in the kidney, leading to the principal cells in the collecting duct to increase water absorption. ADH, antidiuretic hormone; AQP2, aquaporin-2 receptors.

ADH, also known as arginine vasopressin (AVP), regulates water homeostasis in the body. Normally, ADH binds to vasopressin receptor 2 (V2) in the kidney tubule and leads to activation and upregulation of aquaporin 2 (AQP-2) channels as shown in **Figure 1**. When this complex mechanism of autoregulation does not occur properly, urine cannot be concentrated (5).

DI is separated into two distinct disease processes: central and nephrogenic DI.

1. Central diabetes insipidus (CDI): characterized by deficient ADH secretion from the posterior pituitary.
2. Nephrogenic diabetes insipidus (NDI): characterized by deficient V2 or aquaporin-2 response in the kidneys in the setting of appropriate ADH levels.

The rarity of DI perpetuates unfamiliarity with presentation, diagnosis, and treatment of this disease. This article will review presentation, diagnosis, and treatment of DI.

## 2 Causes of diabetes insipidus

CDI is most often acquired, and up to 36% of cases are due to head trauma or transfrontal/transsphenoidal surgery when the hypothalamus or pituitary gland is disrupted. CDI can present in varied ways in response to trauma or surgery. The most common presentation is a temporary disruption in ADH within 24 hours of the brain insult that resolves within a few days. Alternatively, another presentation CDI that remains permanent. The final presentation after brain insult is a triphasic response to complete pituitary stalk resection. This involves axon shock with CDI physiology lasting up to a few days, an antidiuretic phase with unregulated ADH release lasting up to two weeks, and a final phase of recurrence of DI after all the ADH stores have been released (1). Other CDI causes include infiltrative diseases, tumors, inflammatory processes, or autoimmune disorders as outlined in **Table 1**. Though CDI is infrequently due to inherited genetics, more than 55 different mutations that cause a defective prohormone and subsequent ADH deficiency have been identified to cause familial CDI (6).

NDI is frequently caused by medication effect that decreases AQP2 expression. The most common medication to induce NDI is lithium. NDI develops in 40-55% of patients treated with lithium and can present as soon as eight weeks after treatment initiation (2). Though the majority of NDI is induced by medications affecting AQP2 expression, NDI is also linked to various genetic mutations that directly impact the AQP2 gene (7).

Both CDI and NDI have an extensive list of acquired and genetic etiologies, as outlined in **Tables 1**, **2**.

**Table 1:** Causes of Central Diabetes Insipidus (1, 2, 6, 8).

Tumor (10-15%)	Craniopharyngioma Germinoma Meningioma Pituitary Hypothalamic
Trauma (10-25%)	Transfrontal/transsphenoidal surgery Brain injury
Vascular	Hypothalamic infarction/hemorrhage Cerebral infarction/hemorrhage Cerebral aneurysm Sheehan’s syndrome Sickle cell disease
Autoimmune/Inflammatory	Lymphocytic hypophysitis Systemic lupus erythematosus Scleroderma Granulomatosis with polyangiitis IgG4 disease Xanthogranulomatous hypophysitis Anti-vasopressin neuron antibodies Guillain-Barré syndrome
Granulomatous	Sarcoidosis Granulomatous hypophysitis Langerhans’ cell histiocytosis Erdheim-Chester disease
Drug/Toxin	Phenytoin Ethyl alcohol Tetrodotoxin Snake venom
Infections	Meningitis Encephalitis Tuberculosis Toxoplasmosis Cerebral abscess
Congenital/Genetic	Autosomal dominant AVP-neurophysin II gene mutations Wolfram (DIDMOAD) syndrome X-linked recessive defects with subnormal ADH levels

**Table 2:** Causes of Nephrogenic Diabetes Insipidus (1, 2, 7, 8).

Drugs	Lithium Cidofovir Foscarnet Demeclocycline Cisplatin Amphotericin B
Vascular	Sickle cell Acute tubular necrosis
Metabolic	Severe hypokalemia Severe hypercalcemia Starvation and low protein intake
Diseases Affecting the Renal Medulla	Sarcoidosis Amyloidosis Sjogren’s syndrome Multiple myeloma Cystic kidney disease
Congenital/Genetic	Vasopressin V2 Receptor gene mutations Aquaporin-2 gene mutation

## 3 Clinical presentation

Polyuria, polydipsia, and nocturia are the most common presenting symptoms of DI. Polyuria is defined as urine output that exceeds 3 L/day in adults, 2 L/m2 in children, or 50 mL/kg body weight in 24 hours (1, 2). Polydipsia is defined as fluid intake that exceeds 3 liters in 24 hours (2). Symptoms of dehydration and hypernatremia may develop and include weakness, lethargy, fatigue, and myalgias. A clinician should consider DI when a patient presents with such symptoms, but patients may not mention these symptoms in a routine office visit if their quality of life is not heavily impacted. This will delay the diagnosis until a patient overtly discusses the polyuria and polydipsia concerns. Pediatric patients often present with severe dehydration, vomiting, constipation, failure to thrive, and growth retardation. Autosomal dominant DI often presents prior to the age of 10, but the presentation timeline varies widely based on the severity of the ADH deficiency and can present as early as infancy (6). Often partial CDI or NDI will present later in life due to the decreased severity of symptoms (2).

## 4 Diagnosis

The differential when evaluating a patient with polyuria and polydipsia includes DI, primary polydipsia, solute diuresis (uncontrolled diabetes mellitus, recovery from renal failure, administration of high sodium load, mannitol, radiocontrast dye, salt-wasting nephropathies), and appropriate diuretic response (1, 2). A detailed history including dietary and fluid intake, medication reconciliation, head trauma or surgery, headache or visual changes, presence or absence of thirst, and family history will help to elucidate the etiology of polyuria and polydipsia. Diagnostic testing, however, is critical to confirm hypotonic polyuria and distinguish between CDI, NDI, and primary polydipsia. After hypotonic polyuria has been diagnosed and causes of solute diuresis have been ruled out, the patient should be referred to a specialist for further testing.

When DI is suspected, a 24-hour urine collection should be obtained to confirm hypotonic polyuria (8, 9). Hypotonic urine is defined as a urine osmolality less than 300 mOsm/kg. Polyuria is defined under “Clinical Presentation.” After confirming hypotonic polyuria, further information is needed to distinguish DI from primary polydipsia (8). A way to help differentiate primary polydipsia from DI is assessment of serum sodium. An elevated serum sodium >147 mmol/L is diagnostic of DI in the setting of hypotonic polyuria, while a low serum sodium <135 mmol/L is diagnostic of primary polydipsia (10). However, most patients with DI will present with serum sodium levels between 136-147 mmol/L since they are drinking to thirst and will require further diagnostic testing.

The gold standard diagnostic test for DI is an indirect water deprivation test. The premise behind this test is that when healthy patients are deprived of water, their bodies will appropriately concentrate urine to above 800 mOsm/kg. When patients with DI are deprived of water, the kidneys remain unable to concentrate urine and will continue to have hypotonic polyuria with a urine osmolality under 300 mOsm/kg. This is usually performed in a hospital setting or an endocrine testing center due to the need for close monitoring and medical supervision with labs every few hours during the test. During the water deprivation test, the patient’s water intake is restricted until one of these end points is met: 17 hours duration, plasma sodium concentration 150 mmol/L or greater, a loss of 3-5% of patient’s body weight, two consecutive urine osmolality measures do not differ by >10% and loss of 2% body weight, or orthostatic hypotension (1). At the test conclusion, the diagnosis of DI can be made if polyuria persists with water deprivation and urine osmolality remains <300 mOsm/kg. In primary polydipsia or normal renal function, the urine osmolality should rise to >800 mOsm/kg (10). The sensitivity and specificity of the water deprivation test varies depending on the specific protocol used and the population being tested. There are several reasons why the classic water deprivation can exhibit poor diagnostic outcomes. First, chronic polyuria can impact kidney concentration capacity through renal washout or downregulation of renal expression of AQP2 water channels, which decreases the renal response to desmopressin or osmotic stimulation. Second, patients with CDI and impaired glomerular function may also have a higher urine concentration than expected due to compensatory increase expression in the AVPR2 gene. A final confounding scenario occurs in acquired NDI often present with only a partial resistance to ADH, which would appear clinically similar to partial CDI (1). A review article reported that the sensitivity of the water deprivation test ranges from 80% to 100%, while the specificity ranges from 67% to 100%. Other studies have reported similar findings (11).

The water deprivation test is often followed by an administration of desmopressin (DDAVP) (2-4 mcg subcutaneously or intravenously (intranasal is not preferred due to unpredictable absorption) to differentiate central vs. nephrogenic DI. After desmopressin is given, an increase in the urine osmolality >50% is consistent with complete central DI and to a lesser degree in partial CDI. No or minimal increase (<15%) in urine osmolality after desmopressin is consistent with complete NDI. Partial NDI can have an up to 45% increase in urine osmolality (12). The desmopressin test has a sensitivity of 97-100% and a specificity of 80-100% for distinguishing between CDI and NDI (13).

**Figure 2:**
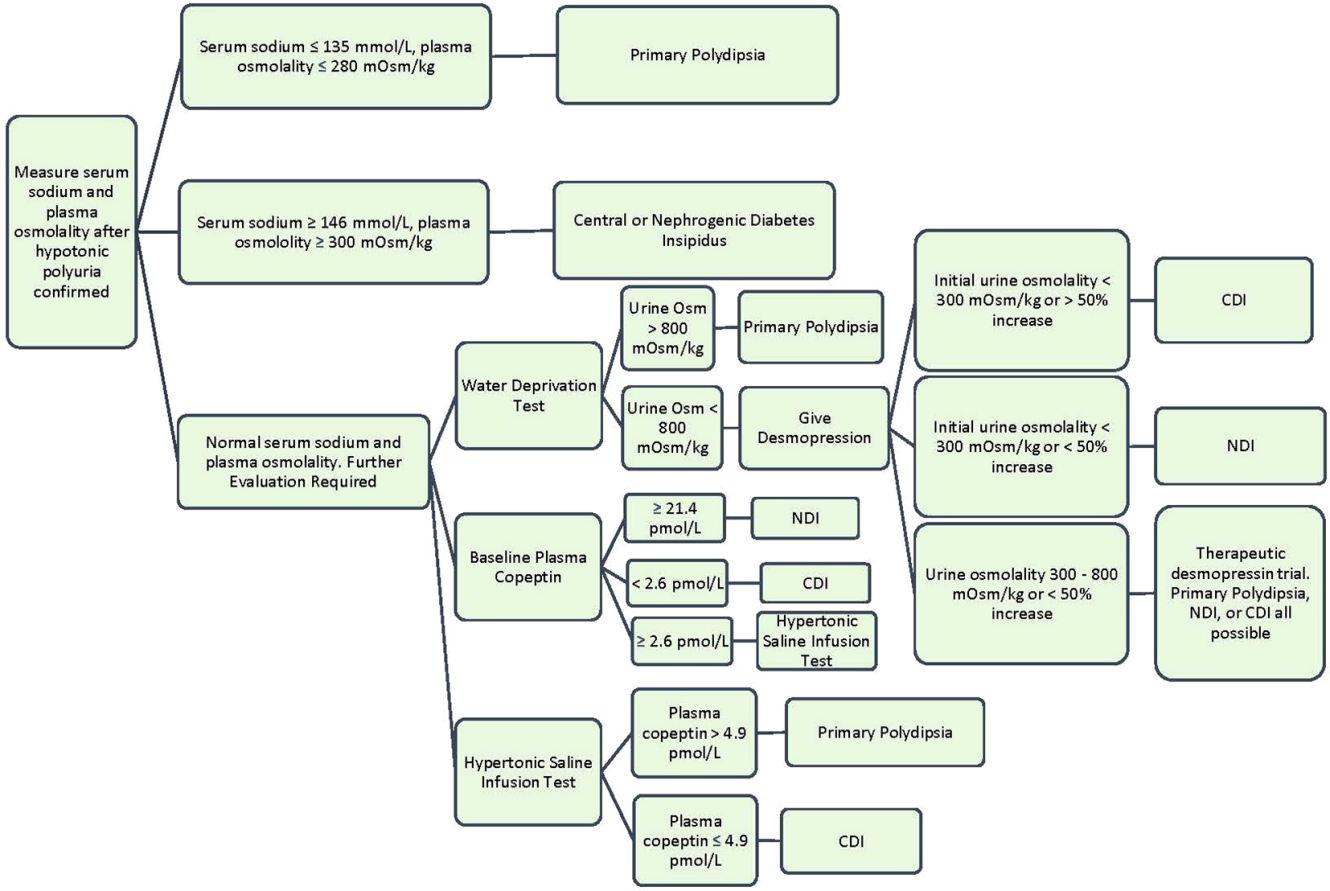
Diagnostic Algorithm for Hypotonic Polyuria Syndromes (1, 5, 8).

When urine osmolality is between 300 – 800 mOsm/kg after water deprivation, the differential includes partial DI or primary polydipsia. This intermediate urine osmolality supports some endogenous ADH activity or ADH response. In partial DI, there is <50% increase in urine osmolality after desmopressin administration (8). This algorithm is visualized further in **Figure 2**.

### 4.1 Copeptin measurement

Due to partial DI and longstanding primary polydipsia, the water deprivation test has diagnostic limitations. It also requires either an inpatient admission or intensive clinical support in the outpatient setting. Copeptin is a new and promising clinical diagnostic marker from the blood that can aid in the diagnosis of DI. Copeptin is the C-terminal peptide of pro-vasopressin and is co-secreted with ADH from the posterior pituitary (5). The plasma levels of copeptin strongly correlate with plasma ADH and can serve as a surrogate marker. ADH is short lived in the blood stream and difficult to measure, whereas copeptin is stable for days after a blood sample is collected (1). Copeptin can be measured in commercially available blood assay, but it is not yet routinely used clinically and not readily available at many institutions.

Copeptin can be measured at baseline, after hypertonic saline infusion, and after desmopressin administration to help with diagnosis (10). A baseline copeptin level of >21.4 pmol/L diagnoses NDI with 100% sensitivity and 100% specificity (8). Levels less than this require osmotic stimulation to bring the sodium level above 150 mmol/l, which is accomplished by hypertonic saline infusion. After osmotic stimulation, a copeptin level below 4.9 pmol/L is diagnostic of central DI, while a level ≥4.9 pmol/l is diagnostic of primary polydipsia. A multicenter prospective trial evaluated measuring copeptin levels after osmotic stimulation to distinguish CDI from primary polydipsia, and it showed this method to have a diagnostic accuracy of 96.5% (93.2% sensitivity and 100% specificity) (14).

## 5 Treatment

### 5.1 General considerations

A mainstay of DI treatment, whether CDI or NDI, is adequate access to water. Allowing the patient to drink to thirst is vital to allow the patient to balance out the water loss experienced with DI. In cases of mild DI, replacing the free water deficit through oral fluids alone can be a suitable treatment. Not every patient needs specific targeted therapies, especially in mild cases (15, 16). In instances where the thirst mechanism is impaired (acute illness, altered cognition, vomiting, respiratory failure), patients should be admitted to the hospital to treat the hypernatremic dehydration that ultimately develops from the inability to keep up with oral intake. Endocrinology consult should be sought in these cases. In severe cases of DI or cases in which symptoms are not controlled with fluid intake alone, there are specific treatment options as discussed below. Signs that patients are incapable of keeping up with oral fluid alone include high sodium on routine lab draws, altered consciousness, and lack of thirst. Patients with adequate oral intake experiencing poor quality of life from nocturia, polyuria, and interrupted sleep cycle would also benefit from additional treatment. It is also possible for high fluid intake to be harmful, which has been demonstrated by children developing hydronephrosis, hydroureter, and fluorosis from excessive fluid intake (17). If it is unclear which treatment is best suited for the patient, endocrinology consult should be placed.

### 5.2 Treatment of central diabetes insipidus

#### 5.2.1 Desmopressin

The most common treatment for CDI is synthetic vasopressin (desmopressin or DDAVP) (18). Desmopressin became available in the 1970s and has remained the main treatment for CDI (15). Desmopressin is an analog of ADH but with a few modifications. The cysteine group has been removed to prolong its half-life, and the d-arginine has been replaced with l‐arginine, which eliminates its pressor activity (16). There are many different forms of desmopressin available and outlined in **Table 3**, but the first line options are traditionally the oral or intranasal formulations. Most patients prefer the oral form for convenience. The disadvantages of the nasal form are that it needs to be refrigerated and has reduced effectiveness if nasal mucosal inflammation is present (16).

**Table 3:** Desmopressin Formulations for Treatment of Diabetes Insipidus.

Forms	Doses	Considerations
Desmopressin acetate tablets	0.1mg 0.2mg	• Recommended starting dose 0.05mg orally one or two times a day • Usual dose range 0.1mg to 1.2mg divided 2-3 doses (19)
Desmopressin acetate Nasal spray	10mcg per 0.1mL spray	• 4 years and older • Usual dose 10-40mcg daily single dose or divided dose • Peds- 10mcg to 30mcg (20)
Desmopressin acetate Sublingual tablets	Women - 27.7mcg daily Men - 55.3mcg daily	• Given one hour before bed • Limit water 1 hour before and 8 hours after administration (21)
Desmopressin Acetate – subcutaneous/intravenous injection	1-2mcg twice a day	• Used in Central DI for management of temporary polyuria and polydipsia following head trauma or surgery (22)

Patients are typically started on a low dose of desmopressin at night to control nocturia (16). Response to this treatment is monitored clinically, and the dose is slowly increased until symptoms are controlled. The medication starts to work within an hour and lasts for six to eight hours. Clinical response varies between individuals, especially when considering age and gender. The dose requires weekly adjustments until there is stability over a month period to ensure an appropriate dose is maintained (15).

When starting desmopressin, a common complication is development of hyponatremia (16). After desmopressin administration, water intake that exceeds the physiologic need is no longer being lost though the kidneys. This accumulation of excess water in the body causes hyponatremia, most often euvolemic (16). As a result, it is recommended to allow aquaresis to occur, which is the excretion of water without losing electrolytes. This should counteract the development of hyponatremia, but the desmopressin dose should be lowered if hyponatremia persists despite having adequate aquaresis. Patients can be given different options on how to perform aquaresis to remove any excess fluid:

1. Delay a dose of desmopressin once or twice a week until two to three voids (aquaresis) are achieved, then take the next dose of desmopressin.
2. Delay each dose of desmopressin until there is slight aquaresis and then take the medication. This is extremely effective as there is constant renal clearance of excess water.
3. Omit one tablet once a week. This is also effective but will cause polyuria for the day.

Desmopressin is the main treatment for central diabetes insipidus, but in some places where desmopressin is not available, other medications are used including thiazide diuretics, carbamazepine, chlorpropamide, clofibrate, and indapamide (18). Desmopressin remains the mainstay of treatment as these alternatives carry their own list of side effects.

#### 5.2.2 Thiazide diuretics

Of the second line agents, thiazide diuretics deserve mention as they are often used in a special population of infants and young children. They work by decreasing distal tubal reabsorption of sodium by inhibiting the Na-Cl cotransporter. This leads to natriuresis and volume contraction which then results in increased water and sodium reabsorption in the proximal tubule (17). This is a common treatment used in infants with central diabetes insipidus whose diet is liquid based (23). In a study done on infants treated with thiazide, it was shown that they were safe and effective in infants and young children (23). The general recommendation is that as the patient ages and transitions to primarily solid food, treatment is transitioned to desmopressin (17).

### 5.3 Treatment of nephrogenic diabetes insipidus

#### 5.3.1 General considerations

Acquired NDI should be treated by reversing the underlying cause, if possible. Lithium-induced NDI may be reversible upon cessation of lithium therapy, with AQP-2 expression increasing in 1 to 2 weeks after stopping lithium in rat models (24). Clinically, symptoms may resolve as soon as 3 weeks after cessation in some patients. However, many patients may never return to baseline urinary concentration as demonstrated in a study showing that 17 of 27 (63%) patients had a concentrating capacity under 800 mOsm/kg 1 year after lithium cessation (25). Prolonged use of lithium will predispose to irreversible NDI, though the precise length of time for this is not well-established (26). Other reversible causes of NDI include hypokalemia, hypercalcemia, and obstructive uropathy (27).

Since the reversal of renal resistance to ADH is often not possible due to long-standing tubular damage or congenital mutations, the mainstay of treatment is adequate water intake to avoid dehydration and hypernatremia. This may require significant fluid intake, which can lead to bothersome polyuria, nocturia, interrupted sleep cycle, and poor quality of life. High fluid intake can also be challenging in children and infants; growth failure may require placement of a gastrostomy tube (28, 29). Further management then aims to reduce urine output, which may be achieved to some extent with low-solute diets, thiazide diuretics, potassium-sparing diuretics, and prostaglandin synthesis inhibitors (30). For patients with NDI that impairs daily activities of living, endocrinology and nephrology should be involved for consideration of other therapies.

#### 5.3.2 Diet

Minimizing osmotic load is recommended in patients with NDI. Restricting the amount of protein and sodium ingested will in turn decrease the amount of protein metabolites and sodium excreted. This has been shown to cause reduction in urinary frequency (31). Ideally, protein intake is limited to no more than 1.0 g/kg and sodium no more than 2.3 g/day (less than 100 mEq/day), and some authors recommend a target osmotic load of 15 mOsm/kg/day (32–34). Such restriction in pediatric patients may compromise normal growth and development, making dietary management in this population challenging.

#### 5.3.3 Diuretics

Thiazides are well known to help in CDI, and studies show some benefit in NDI as well. An early study in patients with NDI showed treatment with hydrochlorothiazide combined with a standard diet containing 9 mEq of sodium per day led to a decrease in urine output to about 50% of baseline after 3 days (35). Hydrochlorothiazide may be initiated at 1-4 mg/kg/d divided into 1-2 daily doses. Chlorothiazide is available in liquid form, making it an attractive option for infants (17).

#### 5.3.4 Desmopressin

Patients with partial NDI may respond to supraphysiologic doses of desmopressin. A nationwide survey in Japan revealed that patients with congenital NDI were treated with DDAVP therapy, which was effective in about 25% of patients (30). While DDAVP can be considered in a patient with partial NDI, there is no consensus when to trial high dose DDAVP. It is generally reserved for patients with inadequate response or contraindications to diuretics. Case reports have described reduction in urine output in both acquired (36–41) and congenital partial NDI (42, 43) with DDAVP doses as high as 80μg intravenously 3-4 times/day, 20μg intranasally 4 times/day, and 720μg/day orally.

#### 5.3.5 Novel therapies

There are multiple novel therapies currently being researched to improve the treatment of NDI. In approximately 90% of patients with congenital NDI, mutations in the *AVPR2* gene cause misfolding of AVPR2 and its entrapment in the endoplasmic reticulum (ER) (44). Perhaps the most studied therapeutic strategy to treat congenital NDI is to salvage these receptors from the ER via molecular chaperones. Nonpeptide AVPR2 antagonists, or vaptans, can bind to the mutant AVPR2 at the AVH binding site, induce proper folding, and allow for trafficking to the cell membrane. The tighter the bond, the better the protein can escape to the cell membrane. However, the tighter the bond, the less likely the AVPR2 antagonist will dissociate from the receptor and allow for normal AVP (ADH) signaling. Discovering a safe and efficacious AVPR2 antagonist has proven difficult to date. One such AVPR2 antagonist, SR49059 (relcovaptan), moderately reduced urine output in five patients with X-linked NDI, proving the potential of this therapeutic strategy (45). Further clinical development of SR49059 was unfortunately halted due to possible interference with the cytochrome P450 metabolic pathway (45). Alternatively, researchers have investigated AVPR2 agonists, which may have the advantage of directly activating the receptor. AVPR2 agonists have been shown to activate various mutant AVPR2 proteins in the ER and induce AQP2 translocation to the apical membrane (46). Other AVPR2 agonists have been shown to rescue various AVPR2 mutants and restore functional ADH-dependent cyclic adenosine monophosphate (cAMP) signaling (47).

Researchers have investigated bypassing AVPR2 signaling altogether and induce AQP2 expression at the apical plasma membrane. Once strategy to achieve this is via G-protein-coupled receptor activation to increase intracellular cAMP. Indeed, principal cells express prostaglandin E2 receptors (EP2 and EP4), a beta-3 adrenergic receptor, and a secretin receptor. Activation of these receptors has been shown to increase urine osmolality in animal models of NDI (48). To date, no clinical data has been reported regarding these targets.

Cyclic guanosine monophosphate (cGMP) has been shown to increase AQP2 membrane translocation *in vitro* and in animal studies (49). There may be a role for agents that increase intracellular cGMP such as cGMP phosphodiesterase inhibitors. Sildenafil has been shown in a single case report to dramatically increase urine osmolality in a young male with X-linked NDI (50).

Ongoing studies are looking into statins, which are associated with lower risk of NDI in lithium users, and activators of adenosine monophosphate-activated protein kinase (AMPK), which is known to phosphorylate AQP2 (51, 52). Additionally, metformin is a known AMPK activator (53). A study in 2021 evaluated metformin’s effect of urinary osmolality and diuresis in patients with NDI, but it was stopped due to lack of effect (54). All of these drug classes have been shown to increase AQP2 expression in principal cells and increase urine output in animal models of congenital NDI (52, 55–57). Unfortunately, at this time, published studies on NDI treatment in humans are scarce and limited to a few case reports or studies including very few participants.

### 5.4 Treatment of diabetes insipidus in the hospital setting

Hospital admission is indicated in patients experiencing hypernatremic dehydration as a result of inadequate fluid intake. Excessive water loss and severe hypernatremia can lead to cerebral dehydration and brain shrinkage. When occurring slowly, the brain has time to adapt and minimize long-term complications. Patients may experience non-specific symptoms of vomiting and nausea that can progress to lethargy and altered mental status. On the other hand, the brain does not have time to compensate in the setting of abrupt tonicity changes with acute, severe hypernatremia. In these cases, complications of acute brain shrinkage include intracranial bleeding, convulsions, or coma (58). Endocrinology consult should be sought for patients admitted to the hospital. In the absence of hypovolemic shock, treatment of hypernatremic dehydration requires infusion of hypotonic intravenous fluids. The fluid usually used is 5% dextrose in water since fluid with osmolality greater than urinary osmolality, such as one-half isotonic saline, can worsen hypernatremia in DI.

If hypernatremia develops acutely, defined as being present for 48 hours or less, the goal is to lower the sodium concentration by 1-2 mEq/L/hr and restore normal serum sodium in less than 24 hours. Typically, 5% dextrose in water is infused intravenously rapidly at a rate of 3-6 mL/kg/hour. Rapid correction of sodium in the setting of acute hypernatremia has not been shown to cause harm (58). In patients with renal insufficiency, hemodialysis has been safely used (59).

If hypernatremia develops chronically, defined as being present for longer than 48 hours, cells will have upregulated their intracellular osmolytes to maintain normal solute concentrations (60, 61). In this setting, rapid correction should be avoided as it can lead to cerebral edema. Studies in pediatric patients have shown that limiting correction of hypernatremia to no more than 0.5 mEq/L/hr prevents cerebral edema, seizures, and death (62). Thus, the number of hours it will take for ‘safe’ correction of hypernatremia can be estimated by doubling the difference between the starting sodium and goal sodium. Furthermore, the free water deficit is calculated by the following formula:


$$\begin{array}{c} \text{Free}\;\text{Water}\;\text{Deficit}\;\left(\text{L}\right)\;\text{in}\;\text{men}\\ =\text{Total body weight }\left(\text{kg}\right)\times 0.6\;\times \left(Serum\;sodium-140\right)÷140 \end{array}$$

$$\begin{array}{c} \text{Free}\;\text{Water}\;\text{Deficit}\;\left(\text{L}\right)\;\text{in}\;\text{women}\\ =\text{Total body weight }\left(\text{kg}\right)\times 0.5\;\times \left(Serum\;sodium-140\right)÷140 \end{array}$$

Taken together, an appropriate starting hourly rate of infusion can be determined by dividing the free water deficit over the estimated ‘safe’ time for correction in hours. Frequent monitoring of urine output, serum chemistries, and clinical status is vital to optimize outcome. A basic metabolic panel every four hours will allow electrolyte and glucose monitoring. High rates of dextrose and water may lead to osmotic diuresis from glucosuria, which may necessitate insulin therapy or alternative hypotonic fluid such as 2.5% dextrose in water or one-quarter normal saline. The frequency of laboratory monitoring can be decreased as the patient shows clinical stability and improvement. Urine output requires strict monitoring, and there is no specific urine output target. The intravenous fluid rate should be adjusted to slightly exceed the hourly urinary output to ensure the patient receives a positive fluid balance.

In summary, there are many considerations which guide fluid choice and infusion rate such as clinical assessment of volume status, ongoing fluid losses, monitoring for development of hyperglycemia from dextrose in water infusion, and frequent laboratory assessment. When possible, the patient should begin taking fluids orally, such as water or, for infants, milk.

## 6 Conclusion

DI can lead to poor quality of life and even increased mortality for patients, making prompt diagnosis and treatment critical. Due to the rarity of DI, many clinicians may not be familiar with the clinical manifestations, diagnosis, and treatment of this disease process. **Table 4** simplifies the approach to such patients. Measuring urine osmolality after water deprivation remains the mainstay of diagnosis, but there is promising research regarding copeptin as a surrogate ADH marker that may play a higher role in the future of DI diagnosis. ADH plays a critical role in DI. When the posterior pituitary gland cannot secrete ADH, central DI develops. When tubular cells in the collecting duct of the kidneys do not respond to ADH, nephrogenic DI develops. Treatment is focused on improving quality of life in patients as DI cannot be cured. Patients with both central and nephrogenic DI need adequate access to water. ADH replacement with desmopressin is the mainstay of treatment for CDI, while ADH replacement is not effective for patients with NDI. NDI is a complex disorder that may require various treatments, and studies are ongoing for directed treatment for this condition.

**Table 4:** Practice Recommendations (1, 2, 13, 15, 19).

When to suspect DI	• Adult with polyuria, polydipsia, and nocturia • Child with dehydration, vomiting, constipation, failure to thrive, and growth retardation • Often a normal sodium level since patients will drink to thirst
Main Subtypes of DI	• Central DI • Nephrogenic DI
Differential Diagnosis for Polydipsia and Polyuria	• DI • Primary polydipsia • Solute diuresis: uncontrolled diabetes mellitus, recovery from renal failure, administration of high sodium load, mannitol, radiocontrast dye, salt-wasting nephropathies • Appropriate diuretic response
Diagnosis	• Indirect water deprivation test (**A**) • Must exclude other causes of polyuria and polydipsia: primary polydipsia, solute diuresis, appropriate diuretic response (**A**) • See **Figure 2** for further diagnostic approach
Treatment of Central DI	• Desmopressin 0.01-0.02 mg orally or 2-4 mcg intranasally once or twice daily (**A**) • Must monitor for hyponatremia as an adverse effect of desmopressin (**A**)
Treatment of Nephrogenic DI	• Correct the underlying cause, such as stopping medication or correcting electrolyte abnormalities (**B**) • Thiazide diuretics may decrease urine output and increase urinary concentration (**C**)
When to Refer to Endocrinology	• Hospitalization required for complications of DI • Hospitalization for water deprivation test • Diagnosis is unclear • Central DI: Patient is failing first line treatment • Nephrogenic DI: Patient quality of life heavily impacted and interested in further treatment options • Any time a clinician is seeking assistance managing a patient with DI

STRENGTH OF RECOMMENDATION (SOR).

A GOOD-QUALITY PATIENT-ORIENTED EVIDENCE.

B INCONSISTENT OR LIMITED-QUALITY PATIENT-ORIENTED EVIDENCE.

C CONSENSUS, USUAL PRACTICE, OPINION, DISEASE-ORIENTED EVIDENCE, CASE SERIES.
